# Intolerance of Uncertainty and Tendency to Worry as Mediators Between Trust in Institutions and Social Support and Fear of Coronavirus and Consequences of the Pandemic

**DOI:** 10.3389/fpsyg.2021.737188

**Published:** 2021-11-10

**Authors:** Tatjana Vukosavljević Gvozden, Aleksandar Baucal, Ksenija Krstic, Severina Filipović

**Affiliations:** Department of Psychology, Faculty of Philosophy, University of Belgrade, Belgrade, Serbia

**Keywords:** COVID-19 fear, pandemic, intolerance of uncertainty, social support, institutional trust, cognitive behavioral therapy

## Abstract

The aim of this article is to formulate and test a model integrating cognitive behavioral therapy (CBT) findings regarding the importance of intolerance of uncertainty (IU) and tendency to worry with findings regarding the importance of trust in institutions, other people, and social support. We assumed that trust in institutions, other people, and social support reduce fear of the coronavirus and of the consequences of the pandemic in a direct way, but also indirectly by enhancing one’s sense of control and diminishing the tendency to worry which, further, attenuates fear of the coronavirus and fear of the consequences of the pandemic. An online survey was conducted among the Serbian general population (*N* = 1409, 78.1% female, *M* = 38.82, SD = 9.24) at the end of April 2020, which included questionnaires on fear of SARS-CoV-2, fear of consequences of the pandemic, social support, trust in other people and trust in political and health institutions. The model has been validated by data from our study suggesting that it represents one possible pattern of interrelationships between social and intrapsychic variables in the pandemic situation. Results also showed that the COVID-19 related fears are related more strongly to intrapsychic variables – IU and tendency to worry – than to variables operationalizing social context relevant for coping with pandemic at the social and individual level.

## Introduction

The consequences of the COVID-19 pandemic are visible in almost all spheres of life – professional, educational, familial, and social. The pandemic has also had a significant impact on people’s psychological state, particularly a noticeable increase in fear and anxiety (Asmundson and Taylor, 2020a; Lee et al., 2020; Wang et al., 2020). For example, nearly half of Americans are afraid that they might become seriously ill and two-thirds of them are afraid of the potential long-term consequences of the pandemic for the economy (Canady, 2020). Research indicates that the increase in distress is influenced by various factors: previous mental health issues – anxiety disorders in particular (Asmundson et al., 2020; Asmundson and Taylor, 2020b), regular media use, risks for loved ones (Mertens et al., 2020), as well as intolerance of uncertainty (IU) (Bakioğlu et al., 2020; Mertens et al., 2020) and one’s tendency to worry (Baiano et al., 2020). Some studies suggest that being a parent (especially a mother) and being chronically ill (Korajlija and Jokic-Begic, 2020), as well as lack of social support (Cao et al., 2020; Skalski et al., 2020), are also factors associated with an increase of COVID-19 related fears and anxiety.

Although various factors that lead to an increase in anxiety during pandemics are known (Taylor, 2019), it is insufficiently clear how intrapsychic factors are related to interpersonal and social factors. The aim of this article is to formulate and to test a model that would integrate the findings concerning IU and tendency to worry (Boswell et al., 2013; Carleton, 2016a, b; Vander Haegen and Etienne, 2016) with findings regarding social factors that have an influence on anxiety during pandemics (Cheung and Tse, 2008; Kim and Kim, 2018; Taylor, 2019). People are afraid of contagion (Bitan et al., 2020; Lee et al., 2020) and of the consequences of the COVID-19 pandemic as well (Canady, 2020; Korajlija and Jokic-Begic, 2020). Therefore, we used the COVID-19 fear scale (Ahorsu et al., 2020), which was available at the period of research, and we also created a scale to measure a broader scope of fears related to the financial and social consequences of the pandemic.

During a pandemic, we are faced with great uncertainty regarding various threats related to our health, finances, and social relations (Taylor, 2019). Uncertainty is a significant stressor for everybody (Rosen et al., 2013; Zlomke and Jeter, 2014). That is particularly the case for individuals with higher IU and tendency to worry which, in the cognitive behavioral literature, represent some of the most important factors that make a person prone to anxiety (Carleton et al., 2007, 2012; Boswell et al., 2013). IU represents a person’s tendency to negatively interpret and react to uncertain events (Dugas et al., 1998, 2004). It is assumed that IU is a trait-like dispositional characteristic resulting from negative beliefs about uncertainty and its implications (Dugas and Robichaud, 2007). More recently, Carleton (2016a) proposes that IU is an individual’s dispositional incapacity to endure the aversive response triggered by the perceived absence of salient, key, or sufficient information, and sustained by the associated perception of uncertainty.

The construct of IU (Freeston et al., 1994), was derived from working with people with a generalized anxiety disorder (GAD). Yet it may be an intrinsic construct for all anxiety disorders (Carleton et al., 2007) and, even more, a transdiagnostic factor for diverse psychopathology (Carleton et al., 2012; Mahoney and McEvoy, 2012; Einstein, 2014; Carleton, 2016a). The IU is normally distributed throughout clinical and non-clinical portions of the population (Carleton et al., 2012), and it is considered fundamental to human experiences (Carleton, 2016a, b).

According to one of the well-known theoretical models (Dugas et al., 1998) as well as research findings (Freeston et al., 1994; Ladouceur et al., 2000), IU plays a key role in the acquisition and maintenance of worries. Worry has been described as “a chain of thoughts and images, negatively affect-laden and relatively uncontrollable” (Borkovec et al., 1983, p. 10). The reported tendency to worry varies continuously across the normal population (Ruscio et al., 2001). However, individuals who cannot tolerate uncertainty often get stuck in an uncontrollable and unproductive proliferation of catastrophic thoughts along the lines of “What if that happens? And what if…?” (Borkovec et al., 1983). Although worry represents an attempt to deal with danger, it intensifies anxiety by leading an individual toward exaggerated catastrophic interpretations of the probability and severity of a threat (Clark and Beck, 2010).

Within cognitive behavioral therapy (hereinafter CBT), cognition is held to affect emotion, behavior, and physiology through our appraisals of ourselves, others, and the world (Beck, 1976; Beck et al., 1979). For example, a person who believes “It’s terrible not to know what lies ahead” is more prone to become anxious when he is confronted with the unknown than a person who does not have this belief. Additionally, the extent to which we are able to shift our attention away from a focus on threat or loss or the extent to which we get stuck in ruminations or worry affects our reactions too (Nolen–Hoeksema, 1991; Bennett-Levy et al., 2004). Tendency to chain catastrophic thoughts in an unproductive and repetitive way intensifies anxiety and hampers the solution-finding process (Hong, 2007; McLaughlin et al., 2007; Ryum et al., 2017). Bearing that in mind, we predict that IU is related to the fear of the coronavirus and of the consequences of the COVID-19 pandemic directly but also indirectly through one’s tendency to worry about events whose outcomes are uncertain.

The psychological reactions of an individual to a pandemic cannot be observed in isolation from interpersonal and wider social factors. The regulation and resolution of a pandemic depend to a great extent on government policies and measures, activities of other community members as well as the capacity of health institutions to provide adequate services to individuals in a timely manner. Social support and trust in people and institutions represent a significant social resource that influences one’s sense of control and an individual’s perceived ability to solve a problem (Beck et al., 1985; Delhey and Newton, 2003; Torche and Valenzuela, 2011). A lack of trust in social systems leads to a feeling of insecurity (Giddens, 1991) and higher anxiety over personal and public security (Tang et al., 2018). In the context of the SARS crisis (Cheung and Tse, 2008), as well as the MERS crisis (Kim and Kim, 2018), people naturally relied on other people or organizations that could provide information and take action to solve the problem. Yet, the mechanisms through which interpersonal and wider social factors affect peoples’ reactions were not elaborated enough. In order to gain a better understanding of the underlying mechanism, and to formulate hypotheses, we turn our attention to CBT theory.

The CBT has long established the key role of threat appraisal in fear and anxiety. Research into IU, a more newly established construct, has largely concentrated on the contributions of trait IU to anxiety. Yet, several issues remain unclear, including whether IU in anxiety-provoking situations is sufficient in itself – independent of threat appraisal – in eliciting high levels of anxiety (Milne et al., 2019).

The factors which are included in threat appraisal are an estimation of the probability of a dangerous incident occurring and the negativity of its impact, as well as the estimation of the coping resources and available rescue factors (Beck et al., 1985; Salkovskis, 1996). We have presupposed that in the situation of the pandemic there are many external factors that affect the threat appraisal in the community. Trust in political institutions that are responsible for adequately managing the response to the pandemic, trust in health institutions which are responsible for providing health care to those who are in need as well as trust in other people and their willingness to adhere to prevention measures are relevant factors for individual threat appraisal of dangerous incidents. If political institutions manage the pandemic crisis in an effective way and inform citizens timely and properly, if health institutions are capable to ensure adequate health care and if most citizens adhere to recommended protection measures then people will perceive lower threats related to the pandemic, and their fear/anxiety will be lower. Some recent studies have already implicated such a possibility by demonstrating that a lack of trust in the government is related to a higher acceptance of conspiracy theories (Bruder and Kunert, 2020) that is by itself related to the higher fear/anxiety related to COVID-19 (Alper et al., 2020; Larsen et al., 2020).

Furthermore, we have presupposed that social factors may allay pandemic related fears and anxiety indirectly by enhancing one’s sense of control. To understand the onset of fear and anxiety, the perception of control over stimuli that signal danger is essential (Chorpita and Barlow, 1998). An individual who is faced with unknowns would feel the least threatened when unknowns are encountered in sufficiently controllable contexts (Carleton, 2016a). Therefore, two persons with the same level of IU and tendency to worry, who have different levels of trust in the capacity of institutions and other people to provide protection from a pandemic, will experience different levels of fear/anxiety related to the pandemic. So, if a person believes that relevant social institutions are reliable and able to handle the various threats, the person will perceive the situation, although being serious, as being under some control. In that case, there are fewer triggers that can activate IU and, consequently, the fears of coronavirus and other pandemic related fears may be less intensive. On the other hand, a person who does not have confidence in the social institutions would perceive far more threats which would activate IU, making the fears of coronavirus and the pandemic related fears more intensive.

**FIGURE 1:**
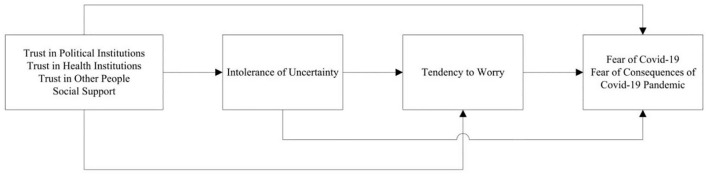
Simplified representation of the initial model of associations between psychosocial and cognitive variables and COVID-19 related anxiety and worry.

Therefore, our study assumes that the COVID-19 pandemic provides an opportunity to understand better the interplay between relevant intrapsychic and social factors and their relation to fears related to the pandemic. The main aim of this study was to test a model including both intrapsychic and social factors relevant for understanding fears of the coronavirus and of the consequences of the COVID-19 pandemic (see Figure 1).

In the study, we are going to test two main hypotheses. The first one is focused on the intrapsychic level, and the second one is focused on the relationship between relevant social factors and pandemic related fear and anxiety:

1. The IU is related to the fear of the coronavirus and of the consequences of the COVID-19 pandemic directly and indirectly through one’s tendency to worry about events whose outcomes are uncertain. This hypothesis is based on results from previous studies, presented above, showing that the IU and the tendency to worry about events whose outcomes are uncertain are important individual factors influencing one’s fear and anxiety related to a given event.
2. Personal relationships and trust in other people and social institutions relevant for coping with the COVID-19 pandemic (trust in political and health institutions and in other people as well as social support from others) can lead to allaying these fears directly by decreasing the level of threats, but also indirectly by enhancing one’s sense of control and diminishing tendency to worry which, further, attenuate fears of contagion and of the consequences of the pandemic. This hypothesis is based on the assumption introduced above that in the situation of a pandemic, the person is aware that she/he needs to rely on social institutions and others to cope with the pandemic and that these social factors decrease or increase perceived risk and uncertainty.

## Materials and Methods

### Sample and Procedure

The sample was obtained by means of online questionnaires distributed between April 25th and May 4th, 2020. During the given period, a state of emergency was in force in Serbia and a curfew was introduced due to the COVID-19 pandemic. Consequently, an invitation to participate was shared on social media. Participation was voluntary and participants were not paid for their contribution. The sample included 1,409 adult participants, of whom 1,101 were women (78.1%) and 308 men (21.9%). Participants were aged between 19 and 65 (*M* = 38.82, SD = 9.24). While completing our survey, 119 participants (8.4%) were infected by COVID-19.

### Instruments

#### Trust in Other People

This construct is measured by three items gauging social trust that are part of the Round 9 of the European Social Survey (ESS Round 9: European Social Survey [ESS-9], 2020). Participants provided their answers on a scale from 0 to 10 where 0 indicates a high level of distrust and 10 indicates a high level of trust (e.g., “One can never be too careful in dealing with other people” vs. “You can trust most people”). Exploratory factor analysis (EFA) showed that all three questions measure the same latent dimension labeled as trust in other people with the first component explaining 73% of the variance. Individual scores are calculated as factor scores (*M* = 0, SD = 1) to get standardized scores, using a regression method. The composite reliability coefficient for the component was 0.82.

#### Trust in Social Institutions and Satisfaction With the Way They Handle the COVID-19 Crisis

Similarly, to the ESS study (ESS Round 9: European Social Survey [ESS-9], 2020), participants were asked to express their level of trust in seven institutions and actors playing an important role in the handling of COVID-19 crisis in Serbia (Government, local authorities, National COVID-19 Response Team, the media, health institutions, doctors and other medical staff, and their own GPs), as well as their satisfaction with the work of the eight institutions and actors during the crisis (President, Government, local authorities, National COVID-19 Response Team, the media, health institutions, doctors and other medical staff, equipment and procedures in health institutions). Participants provided their answers on a scale from 0 to 10 where 0 indicates complete dissatisfaction and 10 indicates complete satisfaction. EFA showed that trust and satisfaction items related to the same institution or actor are highly correlated, so we included all trust and satisfaction items in the same factor analysis. Based on the EFA results, we concluded that participants’ answers can be explained by two factors, with 72.2% of the variance explained (see Supplementary Material). The first factor was related to trust and satisfaction with political institutions, which we labeled as Trust in political institutions (President, Government, local authorities, media, and National COVID-19 Response Team), and the second one was related to trust and satisfaction with health institutions and actors, which we named Trust in health institutions (doctors and other staff in health institutions, GPs, equipment, and procedures in health institutions). Individual scores in these two scales are calculated as factor scores (*M* = 0, SD = 1), using the regression method. The composite reliability for trust in political institutions was 0.95, and 0.85 for trust in health institutions.

#### Social Support

To measure social support, we have developed six items similar to those measuring social capital in the Personal Social Capital Scale (Chen et al., 2009). Participants were asked to estimate their confidence in getting emotional support from six types of others (partner, parents, relatives, friends, neighbors, and colleagues). Answers were provided on a scale from 0 (not confident at all) to 10 (fully confident), so the higher total score indicated a higher level of perceived social support and vice versa. The Cronbach’s α reliability for the scale attained the value of 0.76.

#### Intolerance of Uncertainty

To measure this construct, we used the Serbian adaptation of the Intolerance of Uncertainty Scale (IUS: Freeston et al., 1994; Serbian adaptation: Mihić et al., 2014). The longer 27-item version of the scale was adapted, and then shortened in accordance with the recommendations by Carleton et al. (2007) and Norton (2005). Items with more than one factor loading, items with loading under 0.35, redundant items, and items determined to measure constructs besides IU were excluded (Mihić et al., 2014). The result was the 11-item Serbian version with a 5-point Likert scale consisting of items 3, 5, 7, 8, 9, 10, 12, 15, 19, 20, and 25 from the original scale (e.g., “Uncertainty makes life intolerable” and “Unforeseen events upset me greatly”) by Freeston et al. (1994). The scale measures the overall level of IU, higher scores indicating greater levels of intolerance, with a possibility of calculating scores for two subscales (prospective and inhibitory uncertainty). We only used the overall score in our study. The Serbian adaptation of the scale was found to have good psychometric properties. Cronbach’s α was 0.84, the 11-item version correlated highly with the 27-item version (*r* = 0.95) and the two measures were similarly correlated with the relevant constructs used to assess convergent, divergent, and predictive validity – for example, anxiety sensitivity, psychological flexibility and depression, anxiety, and stress (Mihić et al., 2014). In our study, Cronbach’s α reached the value of 0.90.

#### Tendency to Worry

The Penn State Worry Questionnaire (PSWQ; Meyer et al., 1990) is used to measure the tendency to worry. The instrument has 16 items with a 5-point Likert scale (e. g., “When I am under pressure, I worry a lot,” and “I worry all the time”), and measures the propensity to worry in an excessive, pathological way, without focusing on particular domains of worry (Turk et al., 2004). Higher scores indicate higher levels of worry and vice versa. PSWQ was found to have very good psychometric properties, with Cronbach’s α values above 0.90 in numerous studies (see Turk et al., 2004). This was also the case in our study, where the resulting Cronbach’s α reliability coefficient had the value of 0.92.

#### Fear of COVID-19

We used the Fear of COVID-19 Scale (FCV-19S; Ahorsu et al., 2020) to measure COVID-19 anxiety. The scale includes seven items aimed at measuring fear of the COVID-19 virus, with an emphasis on emotional and physiological arousal. It is a self-report instrument, with a 5-point Likert scale for each item (1 = “strongly disagree” to 5 = “strongly agree”; e.g., “I am most afraid of coronavirus-19” and “I cannot sleep because I’m worrying about getting coronavirus-19”), with a possible total score range from 7 to 35. The scale has already been used in a number of studies and has been translated into several languages, showing good psychometric properties and Cronbach’s α values from 0.82 to 0.89 (Alyami et al., 2020; Bitan et al., 2020; Sakib et al., 2020; Satici et al., 2020; Soraci et al., 2020; Winter et al., 2020). The examination of the Serbian translation replicated previous findings. EFA revealed one factor structure (see Supplementary Material), explaining 50.18% of the variance and the Cronbach’s α value was 0.83.

#### Fear of Consequences of COVID-19 Pandemic

Since the previously described scale measures fears of COVID-19 but not fears of various consequences of COVID-19 pandemic on people’s lives and functioning, we constructed a scale for that purpose. We first conducted a short qualitative study, where we asked 34 people about their fears regarding the COVID-19 pandemic. Upon analysis of their answers, we were able to categorize the content of their fears into five distinct categories (family finances, job, mental health, social relationships, and overall life in the future). With the consultation of an entire research team to ensure content validity, we designed an item with a 5-point Likert scale for each of the five categories from 1 (“strongly disagree”) to 5 (“strongly agree”), the higher values representing the greater level of fear: I’m afraid that my family’s financial state will be jeopardized by the COVID-19 pandemic; I’m afraid that my job will be jeopardized by the COVID-19 pandemic; I’m afraid that the COVID-19 pandemic will have a bad influence on my psychological state; I’m afraid that the COVID-19 pandemic will have a bad influence on my interpersonal relationships (with partner, family, and friends.); and I’m afraid that the COVID-19 pandemic will permanently change my life for the worse (items were formulated in Serbian language and phrasing presented here are our suggestion for English translation). The items had satisfactory corrected item-total correlations ranging from 0.37 to 0.56. EFA revealed one factor solution explaining 47.52% of the variance, with factor loadings ranging from 0.64 to 0.74. Internal consistency of the scale was shown to be α = 0.72. The total score was calculated by adding the answers for each item, so the possible total score ranged from 5 to 25. The scale was shown to have significant and positive correlation coefficients (see section “Results”) with akin constructs like PSWQ and FCV-19S, which can be interpreted as an indication of satisfactory convergent validity. Although we acknowledge that the scale needs further development, it showed satisfactory properties, so we decided to use it for the purpose of this study, given its exploratory nature. There were no scales measuring fear of consequences of the pandemic available at the beginning of our study, but alternatives were developed later (e.g., Taylor et al., 2020a).

### Data Analysis Strategy

We used IBM SPSS Statistics^®^ and IBM SPSS Amos^®^ software for data analysis. First, the properties of the scale measuring fear of consequences of COVID-19 pandemic were assessed, using EFA and reliability analysis (Cronbach’s α). As a preliminary analysis of the associations between the variables in the study, we calculated Pearson’s correlation coefficients.

To test our main hypotheses, we performed path analysis. We designed and tested a serial mediation model in IBM SPSS Amos^®^ software encompassing both our hypotheses: psychosocial variables (trust-related variables and social support) had an indirect effect on COVID-19 related fears, through their effect on IU as a mediator in the first step, which had a further effect on the tendency to worry (a mediator in the second step), which had a direct effect on the COVID-19 related fears. The model also included direct effects of predictors and IU on the outcome variables. We also allowed correlations between the residuals of the fear of COVID-19 and the fear of consequences of the pandemic, given that these constructs are strongly mutually associated and that their association can be a result of exogenous variables that are not part of our study (e.g., personality traits, temperament, and other individual characteristics). We used maximum likelihood (ML) as an estimation method and bootstrapping procedures (with 1,000 bootstrap samples and 95% bias-corrected confidence intervals) in inferring the significance of estimates and their standard errors.

## Results

Descriptive statistics for the variables used in this study are presented in Table 1. As we can see, there are no extreme values in any direction for most variables, which was to be expected given that we collected our sample among the general population. One exception is that participants in our study showed quite a low level of trust in political institutions.

**TABLE 1:** Descriptive statistics for the variables used in the study.

Variable	*M*	SD	Minimum–maximum
T-POL^a^	27.56	22.71	0–90
T-H^a^	33.25	14.67	0–60
T-PPL^a^	15.12	6.54	0–30
SS	39.44	9.18	6–54
IUS	26.05	8.88	11–55
PSWQ	47.83	13.11	18–80
FCV-19S	13.61	5.57	7–35
CF-C19	12.50	4.58	5–25

*N = 1,409.*

*T-POL, trust in political institutions; T-H, trust in health institutions; T-PPL, trust in other people; SS, social support; IUS, Intolerance of Uncertainty Scale; PSWQ, tendency to worry; FCV-19S, fear of COVID-19; CF-C19, fear of consequences of COVID-19 pandemic.*

*^a^For descriptive purposes only, we calculated total scores of trust in political and health institutions and trust in other people by adding scores on items related to each factor.*

Correlations between the variables are displayed in Table 2. Most of the resulting associations are in accordance with our preliminary assumptions. Psychosocial variables (trust in health institutions, trust in other people, and social support) are positively correlated with each other from a very small positive association (e.g., between trust in political institutions and trust in other people) to a very high positive association (e.g., between trust in political institutions and trust in health institutions). Moreover, the psychosocial variables are mostly negatively correlated with the measures of cognitive and emotional dysfunction to a small extent (significant correlation coefficients are ranged between −0.06 and −0.23). Among the psychosocial variables trust in other people and social support have a somewhat higher negative correlation with the measures of cognitive and emotional dysfunction. On the other hand, the cognitive and emotional variables are moderately to highly correlated with each other and with the measures of COVID-19 related fears which that is in line with theoretical expectations and previous research studies.

**TABLE 2:** Correlations between the variables used in the study.

Variable	1	2	3	4	5	6	7	8
1. T-POL	–							
2. T-H	0.67***	–						
3. T-PPL	0.14***	0.18***	–					
4. SS	0.14***	0.27***	0.41***	–				
5. IUS	0.01^ns^	−0.09***	−0.15***	−0.17***	–			
6. PSWQ	0.02^ns^	−0.07*	−0.19***	−0.19***	0.63***	–		
7. FCV-19S	0.04^ns^	−0.07*	−0.06*	−0.14***	0.41***	0.43***	–	
8. CF-C19	−0.13***	−0.22***	−0.12***	−0.23***	0.38***	0.39***	0.54***	–

*N = 1,409. T-PO, trust in political institutions; T-H, trust in health institutions; T-PPL, trust in other people; SS, social support; IUS, Intolerance of Uncertainty Scale; PSWQ, tendency to worry; FCV-19S, fear of COVID-19; CF-C19, fear of consequences of COVID-19 pandemic.*

*p < 0.05; ***p < 0.001; ^ns^–non-significant.

**FIGURE 2:**
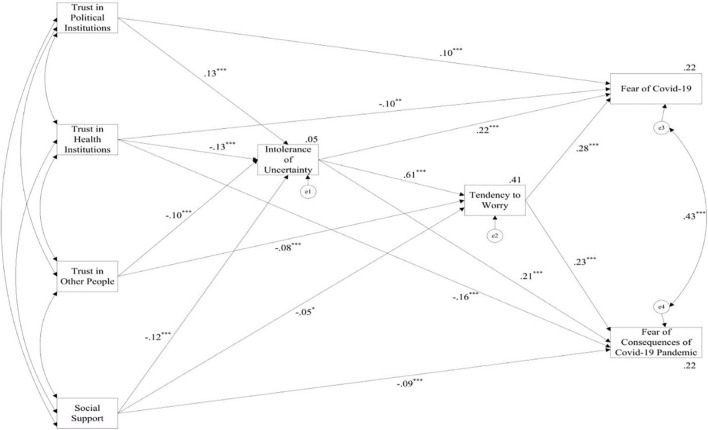
Model of associations between social and cognitive variables and COVID-19 related anxiety and worry. Correlations between independent variables are given in Table 2, thus are not presented here. Path coefficients are standardised regression coefficients. R_2_ values for endogenous variables are presented next to the corner of each variable. e1, e2, e3, and e4 are residuals. *p < 0.05; **p < 0.01; ***p < 0.001.

The serial mediation model (Figure 1) was tested using path analysis. Based on the significance level of the calculated estimates, we trimmed the non-significant paths from the model, in a backward step-by-step procedure. The resulting model with standardized values of direct effects (path coefficients), correlations, and proportion of explained variance is shown in Figure 2. As we can see, the percentage of the explained variance for the fear of COVID-19 and the fear of consequences of the COVID-19 pandemic was 22 and 22.5%, respectively. The percentage of the explained variance of the mediator variables was 4.9% for IU and 41.2% for the tendency to worry.

Values of total effects of the predictors on the mediators and outcomes, and IU on the outcomes, and values of indirect effects of the predictors and IU on the outcomes, with the corresponding standard errors and *p*-values, are shown in Tables 3, 4, respectively.

**TABLE 3:** Standardized total effects (c).

Variable	T-POL		T-H		T-PPL		SS		IUS		PSWQ	
	c	SE	c	SE	c	SE	c	SE	c	SE	c	SE
IUS	0.13**	0.03	−0.13**	0.04	−0.10*	0.03	−0.12**	0.03	–	–	–	–
PSWQ	0.08**	0.02	−0.08**	0.02	−0.14**	0.03	−0.12**	0.03	0.61*	0.02	–	–
FCV-19S	0.15*	0.03	−0.15*	0.04	−0.06**	0.02	−0.06**	0.02	0.39**	0.03	0.29*	0.03
CF-C19	0.05**	0.01	−0.20**	0.03	−0.05**	0.01	−0.15**	0.03	0.35**	0.03	0.23**	0.03

*N = 1,409.*

*SE, bootstrapped standard error of estimate; T-POL, trust in political institutions; T-H, trust in health institutions; T-PPL, trust in other people; SS, social support; IUS, Intolerance of Uncertainty Scale; PSWQ, tendency to worry; FCV-19S, fear of COVID-19; CF-C19, fear of consequences of COVID-19 pandemic.*

Bootstrapped significance (two-tailed): *p < 0.05; **p < 0.01.

**TABLE 4:** Standardized indirect effects (ab).

Variable	T-POL		T-H		T-PPL		SS		IUS	
	ab	SE	ab	SE	ab	SE	ab	SE	ab	SE
PSWQ	0.08**	0.02	−0.08**	0.02	−0.06*	0.02	−0.07**	0.02	–	–
FCV-19S	0.05**	0.01	−0.05**	0.02	−0.06**	0.02	−0.06**	0.02	0.17*	0.02
CF-C19	0.05**	0.01	−0.05**	0.01	−0.05**	0.01	−0.05**	0.01	0.14*	0.02

*N = 1,409.*

*SE, bootstrapped standard error of estimate; T-POL, trust in political institutions; T-H, trust in health institutions; T-PPL, trust in other people; SS, social support; IUS, Intolerance of Uncertainty Scale; PSWQ, tendency to worry; FCV-19S, fear of COVID-19; CF-C19, fear of consequences of COVID-19 pandemic.*

Bootstrapped significance (two-tailed): *p < 0.05; **p < 0.01.

The resulting model had very good fit indices. Non-significant Chi-square was obtained [χ^2^(6) = 9.997, *p* = 0.126], which is rarely the case with large samples, and the value of normed Chi-square was within satisfactory limits (χ^2^/df = 1.663), which indicates a very good overall fit. The other fit indices further supported the adequacy of model fit [GFI = 0.998; AGFI = 0.989; NFI = 0.997; CFI = 0.999; RMSEA = 0.022 with 90% CI (0.004, 0.035); SRMR = 0.015]. We can conclude that the model is consistent with the data, so it might represent a possible pattern of mutual relationships between the variables used in this study.

## Discussion

The aim of the study was to formulate and validate a model depicting potential relationships between the fear of the coronavirus and of the consequences of the COVID-19 pandemic, IU, and the tendency to worry, as well as various social factors. The model assumes that contextual factors (social support as well as trust in institutions and other people) are related to the COVID-19 related fears in a direct way, but also indirectly by enhancing one’s sense of control and diminishing the tendency to worry which, further, attenuate fear of contagion and consequences of the pandemic. The data fits well with the predictions of the model and the two hypotheses formulated based on the model. Based on the final model, it can also be concluded that the COVID-19 related fears are related more strongly to intrapsychic variables – IU and tendency to worry – than to variables operationalizing social context relevant for coping with pandemic at the social and individual level (see Figure 2 and Table 3).

Our findings regarding the connection between IU and the fears of the coronavirus and the consequences of the pandemic are in accordance with numerous earlier findings. It is known that individuals who are intolerant of uncertainty will perceive many more sources of danger in their daily life and have more reactions of hypervigilance when they are faced with uncertain or ambiguous situations (Dugas et al., 2004; Nelson and Shankman, 2011). For those prone to IU, the possibility of negative outcomes triggers biased interpretations of the situation that serve to increase worry and anxiety (Ladouceur et al., 2000; Dugas et al., 2005; Dugas and Robichaud, 2007). Research conducted during the H1N1 virus pandemic indicates a significant positive correlation between IU and fear of the virus (Taha et al., 2014a, b). Recent studies also indicate that the increase in distress concerning coronavirus is influenced by IU (Bakioğlu et al., 2020; Mertens et al., 2020).

However, our results also suggest that IU has an indirect impact on the fears of the coronavirus and consequences of the pandemic by increasing the tendency to worry. According to research findings, IU is a robust predictor of worry (Ladouceur et al., 1997; Koerner and Dugas, 2008; Buhr and Dugas, 2009) and the content of worry typically concerns future events whose outcomes are uncertain (Sibrava and Borkovec, 2006). Individuals prone to worrying may feel like thinking things through, but often get stuck in an uncontrollable and unproductive proliferation of catastrophic thoughts “What if…” During the pandemic one usually has more unknowns than in situations involving individual crisis. Therefore, people tend to worry about numerous things such as health, family, profession, finances, etc., trying to “prepare for” or to “prevent” potential negative outcomes. Yet, a tendency to chain catastrophic interpretations in a repetitive and unproductive way intensifies anxiety and hampers the solution-finding process (e.g., Hong, 2007; McLaughlin et al., 2007; Ryum et al., 2017). In the context of the pandemic, it intensifies one’s fears of the virus and the consequences of the pandemic.

Our findings also highlight the need to direct future research toward identifying additional mechanisms through which IU influences fears during a pandemic. Recent research suggests that the fear of dangerousness of the coronavirus itself, is a part of a broader concept consisting of several interconnected symptoms, including fears about socioeconomic consequences of COVID-19, COVID-19 related xenophobia, compulsive checking and reassurance seeking, and traumatic stress symptoms (Taylor et al., 2020a, b). A link between IU and COVID-19 fear might be found among these symptoms, with emphasis on checking and reassurance seeking. Reassurance seeking and checking make the threat more predictable and controllable (Taylor et al., 2020a, c). However, these strategies can amplify COVID-19 related fears and worries as they expose a person to an even greater number of fear-evoking stimuli. These behaviors could be triggered by one’s IU, as an attempt to reduce uncertainty.

The findings also point out the complex role that social variables might have in the occurrence of fears of the coronavirus and of the consequences of the pandemic. The final model suggests that the social variables included in the model might be related to fears of the coronavirus and of the consequences of the pandemic in a direct and indirect way, although their effects are relatively small. Out of these variables, trust in health institutions has the greatest impact on the fears of the coronavirus and of the consequences of the pandemic directly and indirectly *via* IU. These findings are in accordance with the earlier ones, indicating that in health-threatening situations, an increase in trust in different institutions and organizations is accompanied by a decrease in fears and anxiety (Sapp and Bird, 2003; Tateno and Yokoyama, 2013). When it comes to the pandemic situation, studies conducted during the SARS epidemic in Hong Kong (Cheung and Tse, 2008) and the MERS epidemic in South Korea (Kim and Song, 2017; Kim and Kim, 2018) show that trust in government and health institutions was negatively correlated with fears and anxiety. Our research complements the existing knowledge corpus by indicating that some of these relationships might be indirect ones *via* their relation to relevant intrapsychic variables.

Regarding trust in other people, our findings show that a higher trust in other people is associated with a lower level of fears of the coronavirus and of the consequences of the pandemic solely indirectly through its relation to the IU and the tendency to worry. As previously established, trust in other people (friends, acquaintances, strangers, etc.) is related to lower anxiety levels in various health-threatening situations (Delhey and Newton, 2003; Tang et al., 2016). An explanation of this finding might be related to the fact that persons with a higher trust in other people may perceive a lower level of risks related to the pandemic. In a pandemic like this one, it is important to build a sense of togetherness in the society because when one has trust that other people will behave responsibly, she or he will find it easier to tolerate uncertainty and will worry less, which could reduce levels of fears of the coronavirus and the consequences of the pandemic.

In our study, a higher level of social support is related to a lower level of persons’ fears of the coronavirus and the consequences of the pandemic, which is in accordance with findings from other studies conducted during the COVID-19 pandemic (Cao et al., 2020; Skalski et al., 2020). Furthermore, our results indicate that social support is related to the COVID-19 related fears both directly and indirectly. The finding complements the previous ones indicating a positive effect of the perceived adequacy of social support, availability of coping resources, as well as perceived control on distress (Mirowsky and Ross, 1989; Ross and Mirowsky, 1989). Social support may enhance one’s sense of control and other personal resources and perceived control would, in turn, attenuate distress (Cheung and Tse, 2008).

Contrary to the previously listed social variables that play a role in protecting against COVID-19 related fears, a higher level of trust in political institutions is related to a higher level of fears both directly and indirectly through intrapsychic variables. This is an unexpected finding bearing in mind that, during the pandemic, government and other political institutions tend to play a vital role in lowering health risks and consequences of the pandemic. Yet, this finding is not unique. It has been established that there is a positive correlation between scores on the coronavirus fear scale and satisfaction with President Donald Trump’s response to coronavirus (Lee et al., 2020). This finding can be interpreted in two ways. One hypothesis is that the influence of trust in political institutions depends on the manner in which they deal with the pandemic. Trust in political institutions in some countries will increase negative effects (as is the case in the United States and Serbia), whereas in others it will lead to a decrease in fears of coronavirus (e.g., in Germany – according to Teufel et al., 2020). An alternative hypothesis would be that individuals with a high level of IU tend to trust political institutions more and reduce uncertainty by relying on figures with a high degree of authority and power. Testing these hypotheses would require additional studies that could further clarify these controversial findings. Regardless, this finding stresses the importance of the role the political institutions have in maintaining the psychological wellbeing of their citizens. In an interesting analysis of factors related to mass hysteria, Bagus et al. (2021) propose that biased media coverage, characterized by a focus on the negative news, politicized media where the politicians are used as the source of negative news, the negative news delivered by an authoritative source, and intentional instilling of fear in the population incite anxiety and promote mass panic. Media coverage of the pandemic in Serbia closely resembled this description, so an alternative line of research could examine how these factors are associated with COVID-19 related fears.

To conclude, the results of this study suggest that, during the COVID-19 pandemic, IU, tendency to worry and various social factors play an important role in the occurrence of fears of the coronavirus and the consequences of the pandemic. The study also shows that IU and the tendency to worry as personality traits have a greater influence on the occurrence of fears of the coronavirus and the consequences of the pandemic than social factors. Finally, the findings of the study also make a specific contribution to a better understanding of potential relationships between social factors and fears by indicating that some of these relationships are mediated partially by IU and the tendency to worry.

Based on all findings, it can be concluded that strategies for preserving mental health in situations like the COVID-19 pandemic should be twofold. The first direction should be focused on individual factors. Besides mental health strategies for those with pre-existing anxiety disorders (Asmundson et al., 2020), it is important to formulate strategies for supporting tolerance of threats and uncertainties related to a pandemic. Other areas of action for the preservation of mental health should relate to the level of society. In the pandemic condition it is important to (a) build a sense of social cohesion and cooperation by implementing protective measures aimed at increasing mutual trust between citizens, through the action of various social and political institutions, (b) raise awareness of the important role of mutual support between people who are living together, and (c) inform citizens about capacities of health institutions to provide adequate healthcare to every citizen.

The main limitation of our study is related to the fact we have used a cross-sectional design. Based on that design, we have been able to test our model assuming that social context has direct and indirect effects on intrapsychic variables. However, we have not been able to test an alternative hypothesis assuming that intrapsychic variables might also have an effect on a person’s attitude toward social context. Therefore, in a future study, it would be necessary to include at least two waves of data collection with the same participants in order to be able to identify relationship between intrapsychic variables in the first way and person’s attitude toward social context in the second wave as well as relationship between person’s attitude toward social context in the first wave with intrapsychic variables in the second way.

Furthermore, since we have collected the data during the lock- down, we relied on an online questionnaire distributed by social media. As a consequence, our study might be influenced by the self-selection bias as well as by the fact that the sample consisted of female participants mostly (about 80%). This method enabled us to collect data during the first wave of the COVID-19 pandemic when participants had been faced with the state of emergence and curfew, but our findings need to be interpreted in light of these limitations.

Moreover, it should also be emphasized that our model explained about 22% of the variance of the fears of the coronavirus and the consequences of the COVID-19 pandemic. This implies that there are other important factors, which are not included in our model, that play a significant role in the occurrence of these fears. The finding that the fears of the coronavirus and the consequences of the COVID-19 pandemic are associated positively with each other independently from the variables included in the model, indicates that some additional factors not included in the model also contribute to their positive relationship. These factors might be related to some personal characteristics (e.g., neuroticism as a personality trait and an anxiety trait), so future studies should include additional relevant individual characteristics to explain the association between the COVID-19 related fears.

## Data Availability Statement

The raw data supporting the conclusions of this article will be made available by the authors, without undue reservation.

## Ethics Statement

The studies involving human participants were reviewed and approved by the Institutional Review Board of the Department of Psychology, Faculty of Philosophy, University of Belgrade, Serbia (Dejan Todorović, Iris Žeželj, and Ljiljana Lazarević) – Protocol #2020-25. The patients/participants provided their written informed consent to participate in this study.

## Author Contributions

All authors listed have made a substantial, direct and intellectual contribution to the work, and approved it for publication.

## Conflict of Interest

The authors declare that the research was conducted in the absence of any commercial or financial relationships that could be construed as a potential conflict of interest.

## Publisher’s Note

All claims expressed in this article are solely those of the authors and do not necessarily represent those of their affiliated organizations, or those of the publisher, the editors and the reviewers. Any product that may be evaluated in this article, or claim that may be made by its manufacturer, is not guaranteed or endorsed by the publisher.
