# Supplementary material for: Intolerance of Uncertainty and Tendency to Worry as Mediators Between Trust in Institutions and Social Support and Fear of Coronavirus and Consequences of the Pandemic
Source: Front Psychol. 2021 Nov 10;12:737188. doi: 10.3389/fpsyg.2021.737188 (PMC8631188; doi:10.3389/fpsyg.2021.737188)
Supplement: Supplementary file 1 [file Table_1.DOCX]

*Supplementary Table 1*

*EFA Results for Trust and Satisfaction with Political and Health Institutions*

| Item | T-POL | T-H |
| --- | --- | --- |
| Satisfaction with the Government | **.882** |  |
| Satisfaction with the President | **.862** |  |
| Trust in the Government | **.862** |  |
| Satisfaction with the local authorities | **.812** |  |
| Trust in the local authorities | **.801** |  |
| Satisfaction with the media | **.723** |  |
| Satisfaction with the National COVID-19 Response Team | **.686** | .521 |
| Trust in the National COVID-19 Response Team | **.675** | .529 |
| Trust in the media | **.670** |  |
| Trust in doctors and other medical staff |  | **.884** |
| Satisfaction with doctors and other medical staff |  | **.865** |
| Satisfaction with health institutions | .353 | **.832** |
| Trust in health institutions | .353 | **.821** |
| Trust in one’s GP |  | **.677** |
| Satisfaction with equipment and procedures in health institutions | .539 | **.625** |

*Note*. N= 1409. The extraction method was principal components with Varimax rotation. Factor loadings below .32 were excluded. Loadings belonging to a factor are bolded within that factor. T-POL - trust in political institutions; T-H - trust in health institutions.

*Supplementary Table 2*

*EFA Results for Fear of Consequences of COVID-19 Pandemic Scale*

| Item | Loadings |
| --- | --- |
| I'm afraid that the COVID-19 pandemic will permanently change my life for the worse. | .745 |
| I'm afraid that my family's financial state will be jeopardized by the COVID-19 pandemic. | .699 |
| I'm afraid that the COVID-19 pandemic will have a bad influence on my psychological state. | .694 |
| I'm afraid that the COVID-19 pandemic will have a bad influence on my interpersonal relationships (with partner, family, friends...). | .669 |
| I'm afraid that my job will be jeopardized by the COVID-19 pandemic. | .636 |

*Note*. N= 1409. The extraction method was principal components.
